# Immune Responses of Chickens Infected with Wild Bird-Origin H5N6 Avian Influenza Virus

**DOI:** 10.3389/fmicb.2017.01081

**Published:** 2017-06-20

**Authors:** Shimin Gao, Yinfeng Kang, Runyu Yuan, Haili Ma, Bin Xiang, Zhaoxiong Wang, Xu Dai, Fumin Wang, Jiajie Xiao, Ming Liao, Tao Ren

**Affiliations:** ^1^College of Animal Science and Veterinary Medicine, Shanxi Agriculture UniversityTaigu, China; ^2^College of Veterinary Medicine, Key Laboratory of Zoonosis Prevention and Control of Guangdong Province, South China Agricultural UniversityGuangzhou, China; ^3^State Key Laboratory of Oncology in South China, Collaborative Innovation Center for Cancer Medicine, Department of Experimental Research, Sun Yat-sen University Cancer CenterGuangzhou, China; ^4^Key Laboratory for Repository and Application of Pathogenic Microbiology, Research Center for Pathogens Detection Technology of Emerging Infectious Diseases, Guangdong Provincial Center for Disease Control and PreventionGuangzhou, China; ^5^College of Animal Science, Yangtze UniversityJingzhou, China; ^6^Guangdong Provincial Wildlife Rescue CenterGuangzhou, China

**Keywords:** influenza virus, H5N6, wild birds, pathogenicity, transmissibility, immune response

## Abstract

Since April 2014, new infections of H5N6 avian influenza virus (AIV) in humans and domestic poultry have caused considerable economic losses in the poultry industry and posed an enormous threat to human health worldwide. In previous research using gene sequence and phylogenetic analysis, we reported that H5N6 AIV isolated in February 2015 (ZH283) in Pallas’s sandgrouse was highly similar to that isolated in a human in December 2015 (A/Guangdong/ZQ874/2015), whereas a virus (i.e., SW8) isolated in oriental magpie-robin in 2014 was highly similar to that of A/chicken/Dongguan/2690/2013 (H5N6). However, the pathogenicity, transmissibility, and host immune-related response of chickens infected by those wild bird-origin H5N6 AIVs remain unknown. In response, we examined the viral distribution and mRNA expression profiles of immune-related genes in chickens infected with both viruses. Results showed that the H5N6 AIVs were highly pathogenic to chickens and caused not only systemic infection in multiple tissues, but also 100% mortality within 3–5 days post-infection. Additionally, ZH283 efficiently replicated in all tested tissues and transmitted among chickens more rapidly than SW8. Moreover, quantitative real-time polymerase chain reaction analysis showed that following infection with H5N6, AIVs immune-related genes remained active in a tissue-dependent manner, as well as that ZH283 induced mRNA expression profiles such as *TLR3*, *TLR7*, *IL-6*, *TNF*-α, *IL-1*β, *IL-10*, *IL-8*, and *MHC-II* to a greater extent than SW8 in the tested tissues of infected chickens. Altogether, our findings help to illuminate the pathogenesis and immunologic mechanisms of H5N6 AIVs in chickens.

## Introduction

Since 2003, multiple highly pathogenic avian influenza A (HPAI) H5 subtypes, including H5N1, H5N2, H5N6, and H5N8, have generated severe epidemics and thus not only tremendous economic losses in the domestic poultry industry, but also serious threats to human health worldwide (Jhung and Nelson, 2015). As of October 3, 2016, at least 856 cases of human infection with avian influenza A (H5N1) virus in 16 countries had been reported to the World Health Organization, among which 452 had ended in death, for an apparent case fatality rate of 52.8% (WHO, 2016). As the natural reservoir for avian influenza viruses (AIVs), wild bird populations can be infected by many such viruses, including the H3, H5, and H7 subtypes AIVs, and thus play a critical role in AIV epidemiology and ecology (Claes et al., 2016; Dhingra et al., 2016; Kang et al., 2017). Thus far, results of the phylogenetic analysis of the hemagglutinin (HA) gene have revealed multiple clades and subclades of H5 subtype AIVs. Among them, H5N6 has replaced H5N1 as the dominant subtype in southern China (Bi et al., 2016a), while clade 2.3.4.4 of AIVs is now considered to be the dominant in China (Saito et al., 2015; Claes et al., 2016). Given recent suggestions that clade 2.3.4.4 of AIVs has become increasingly pathogenic to domestic poultry and wild birds (Claes et al., 2016; Sun et al., 2016), AIV virulence is likely affects multiple factors and depends upon both antigenic drift and the AIV-infected strain in the host immunity (Tscherne and Garcia-Sastre, 2011).

An AIV replicates primarily in the respiratory system (Sturm-Ramirez et al., 2004), from where it spreads to the brain and lymphoid tissues by way of infection. Such infection induces batteries of receptors and triggers a signaling cascade that ultimately activates the host’s immune response. As part of that process, for example, the endosomal Toll-like receptor (TLR) 3 and sphingosine-1-phosphate-1 receptor (S1PR1) recognize double-stranded viral RNA released during the uncoating of an internalized virus (Barton, 2007; Teijaro et al., 2011). During AIV infection in mammals, the endosomal TLR 7/8, which recognizes single-stranded viral RNA, can prompt the production of interferon (IFN)-α and IFN-β (Diebold et al., 2004). As MacDonald et al. (2008) have shown, when TLR7/8 are activated by AIV infection in host cells, the recognition of viral RNA results in the secretion of proinflammatory cytokines (e.g., IL-1β, IL-6) and antiviral cytokines (e.g., IFNs). By extension, the expression of proinflammatory cytokines and IFNs influences both viral clearance and the manifestation of clinical symptoms. At the same time, since major histocompatibility complex (MHC) classes I and II antigen presentation molecules used for AIV uptake activated cellular immunity and humoral immunity of B cells (i.e., IFN-γ) and T cells (i.e., CD3+, CD4+, CD8+), MHC molecules likely play a role in activating host innate immune response to AIV infection (Gromme and Neefjes, 2002; Williams et al., 2002).

In China’s Sichuan Province on May 7, 2014, the first-ever fatal case of human infection by a reassortant H5N6 AIV involved a 49-year-old man with a history of exposure to live poultry. To date, 14 additional cases of human infection with the H5N6 virus in China’s Sichuan, Guangdong, Jiangxi, and Yunnan Provinces—10 of which ended in death—documented by the World Health Organization and World Organisation for Animal Health were characterized as posing a potential risk to public health^1^.

In studies conducted during 2014–2015, we performed epidemic surveillance of AIVs among wild birds at nature reserves in southern China, isolated two novel reassortant HPAI H5N6 viruses, and conducted genetic and phylogenetic analyses to elucidate their molecular features (Kang et al., 2017). By extension, in the present study, we investigated the pathogenicity and transmissibility of the viruses in chickens. In addition, to assess the role of the host innate immune response of H5N6-infected chickens, we examined a complex expression profile of pattern recognition receptors (PRRs), proinflammatory cytokines, chemokines, and MHC molecules in the brain, lung, spleen, and bursa of Fabricius.

## Materials and Methods

### Ethics Statement

All animal experiments were conducted in ABSL-3 facilities and in accordance with the guidelines of South China Agricultural University’s Institutional Animal Care and Use Committee. All animal protocols were approved by the Committee on the Ethics of Animal Experiments of the ABSL-3 Committee of South China Agricultural University (approval no. L102012017001K).

### Viruses and Experimental Animals

Two H5N6 viruses—namely, A/oriental magpie-robin/Guangdong/SW8/2014 (SW8) and A/Pallas’s sandgrouse/Guangdong/ZH283/2015 (ZH283)—used in this study were grown and purified three times in Madin–Darby canine kidney cells by standard plaque assay. The stocks of H5N6 viruses were propagated in 9-day-old specific pathogen-free (SPF) chicken eggs at 37°C for 72 h per the procedure (Yuan et al., 2014). Allantoic fluid pooled from multiple eggs was taken for centrifugation for 2 min at 8,000 rpm, from which the supernatant was harvested and subsequently frozen in aliquots at -80°C for further characterization. The 50% egg infectious dose (EID_50_) titer for egg-grown virus was determined by 10-fold serial dilutions and the titration of each virus in 9-day-old SPF eggs using Reed and Muench’s (1938) method. Six-week-old SPF white leghorn chickens (Guangdong Wens Dahuanong Biotechnology Co., Ltd, Yunfu, China) were held in isolator cages with a feeding space of 117 m^3^ throughout the duration of each experiment.

### Pathogenesis and Transmission Experiments of H5N6 Virus in Chickens

*In vivo* pathogenesis studies of wild bird H5N6 influenza viruses were designed as previously described (Zhang et al., 2008, 2009; Pu et al., 2015). In brief, groups of 12 6-week-old SPF chickens were intranasally inoculated with 0.2 mL of 10^5^ EID_50_ of SW8 or ZH283, while a control group of 12 chickens was inoculated with 0.2 mL of phosphate buffered saline (PBS) using the same route. Three days later, six inoculated chickens from each group were humanely euthanized to test for viral replication in lung, kidney, spleen, cecal tonsils, bursa of Fabricius, trachea, pancreas, liver, heart, brain, duodenum, ileum, descending colon, and jejunum tissue. The remaining chickens were observed twice daily, at 8:00 and 20:00, for clinical symptoms, morbidity, and mortality for 14 days according to the protocol provided by the World Organisation for Animal Health^2^.

Direct contact virus transmission experiments in chickens were conducted per the procedure of (Yuan et al., 2014). Briefly, the chickens of inoculated groups (*n* = 6) were intranasally inoculated with 0.2 mL of 10^5^ EID_50_ of either the SW8 or ZH283 virus in a ABSL-3 laboratory, and after 24 h, additional naïve contact groups (*n* = 3) were also intranasally inoculated with 0.2 mL of PBS and placed in physical contact in the same cage to share feed and water with chickens inoculated with the virus. At 3 days post-infection (DPI), three inoculated chickens were humanely euthanized, and target tissues (i.e., brain, lung, spleen, and bursa of Fabricius) were harvested to determine viral titers and for RNA extraction. At 3, 5, 7, 9, and 11 DPI, oropharynx and cloacal swabs samples were collected for the detection of viral shedding and suspended in 1 mL of PBS. All tested tissues and swabs samples were harvested for viral detection and titration in SPF chick embryos. All surviving chickens were euthanized at 14 DPI, and the serum was harvested and tested for seroconversion by hemagglutination inhibition testing using 1% turkey erythrocytes (Stephenson et al., 2004).

### RNA and cDNA Preparation

Total RNA was extracted from the brain, lung, spleen, and bursa of Fabricius of H5N6-inoculated chickens and mock-infected chickens at 3 DPI using the Takara MiniBEST Universal RNA Extraction Kit (Takara Bio Inc., Tokyo, Japan) following the manufacturer’s instructions. Total RNA (1 μg) was reverse-transcribed with the PrimeScript^TM^ II 1st Strand cDNA Synthesis Kit (Takara Bio Inc.) and stored at -20°C for further study.

### Quantitative Real-Time Polymerase Chain Reaction

Quantitative real-time polymerase chain reaction (qRT-PCR) was performed using a FastStart Universal SYBR Green Master kit (Roche Diagnostics, Shanghai, China). qRT-PCR primers (**Table 1**) were designed from published target sequences and previously reported (Adams et al., 2009) with Primer Premier 7.0 software (Premier Biosoft, Palo Alto, CA, United States). qRT-PCR was performed on a LightCycler480 (Roche Applied Science, Mannheim, Germany), the products of which were purified by using a DNA gel extraction kit (Takara Bio Inc., Tokyo, Japan). For the purposes of assay validation, purified products were cloned into pMD19-T and sequenced to verify correct target amplification.

**Table 1:** Quantitative real-time PCR primers used in this study.

Gene	Forward primer (5′3′)	Reverse primer (5′3′)	Product size (bp)	GenBank accession no.
GAPDH	CCTCTCTGGCAAAGTCCAAG	CATCTGCCCATTTGATGTTG	200	NM_204305
TLR3	ACAATGGCAGATTGTAGTCACCT	GCACAATCCTGGTTTCAGTTTAG	123	NM_001011691
TLR7	TGTGATGTGGAAGCCTTTGA	ATTATCTTTGGGCCCCAGTC	218	DQ780342
S1PR1	TTGCCTTGTCAGCTTCTGTG	CGTGGAGCAGTTTGACAAGA	203	XM_422305
IL-1β	GCTCTACATGTCGTGTGTGATGAG	TGTCGATGTCCCGCATGA	80	NM_204524
IL-6	CCTGTTCGCCTTTCAGACCT	GGGATGACCACTTCATCGGG	171	EU170468
IL-8	ATTCAAGATGTGAAGCTGAC	AGGATCTGCAATTAACATGAGG	196	DQ393272
TNF-α	CCGCCCAGTTCAGATGAGTT	GCAACAACCAGCTATGCACC	130	AY765397
IFN-α	ATGCCACCTTCTCTCACGAC	AGGCGCTGTAATCGTTGTCT	387	EU367971
IFN-β	CCTCAACCAGATCCAGCATT	GGATGAGGCTGTGAGAGGAG	259	AY831397
IFN-γ	TGAGCCAGATTGTTTCGATG	CTTGGCCAGGTCCATGATA	248	DQ906156
CCL5	GTTTGGGGCTGATACAACCG	CCTTCACATGATTCTGGGGCA	71	NM_001045832
MHC-I	AAGAAGGGGAAGGGCTACAA	AAGCAGTGCAGGCAAAGAAT	222	NM_001031338
MHC-II	CTCGAGGTCATGATCAGCAA	TGTAAACGTCTCCCCTTTGG	312	DQ008588


### Calculations and Statistical Analysis

The relative expression ratios of target genes in tested tissues vs. those in control tissues were calculated by the 2^-ΔΔCT^ method using the chicken housekeeping gene glyceraldehyde-3-phosphate-dehydrogenase (NM_204305) as the endogenous reference gene in order to normalize the level of target gene expression (Livak and Schmittgen, 2001). Standard deviations were determined by using the relative expression ratios of three replicates for each gene measured. Differences of virus titers and mRNA expression levels were statistically analyzed with an unpaired non-parametric test and paired Student’s *t*-test, respectively, using GraphPad Prism version 6.0 (GraphPad Software Inc., La Jolla, CA, United States) software. Compared to the mock-infected control, *p* < 0.05 and *p* < 0.01 were considered to indicate a statistically significant difference unless stated otherwise.

## Results

### Pathogenesis of Wild Birds Origin A(H5N6) Influenza Viruses in Chickens

In previous research, we characterized the two H5N6 influenza viruses isolated from apparently healthy wild birds in 2014 and 2015 in Guangdong Province, China. On the one hand, SW8 was isolated from an oriental magpie-robin, and its PB2 gene with poultry H5N6 viruses shared the highest nucleotide similarity with that of A/chicken/Dongguan/2690/2013 (H5N6). On the other hand, ZH283 was isolated from a Pallas’s sandgrouse, and its PB2 gene shared the highest nucleotide similarity with that of A/Guangdong/ZQ874/2015 (H5N6) isolated from a 40-year-old woman who reported exposure to domestic poultry^3^.

**FIGURE 1:**
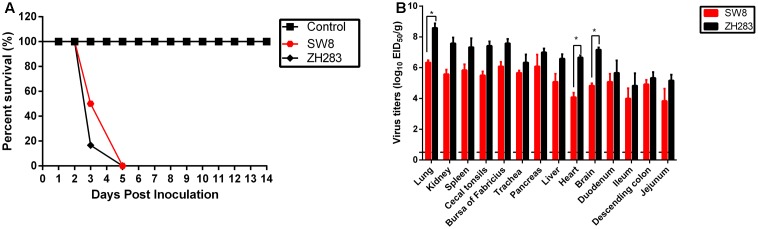
Pathogenesis of H5N6 viruses in specific pathogen-free chickens. **(A)** Percentage of survival of SW8 and ZH283 in chickens. **(B)** Comparison of two A(H5N6) influenza virus titers of wild bird origin in chickens. Groups of 12 6-week-old chickens were intranasally inoculated with 0.2 mL of 10^5^ EID_50_ of SW8, ZH283, or PBS; six chickens in each group were euthanized at 3 days post-infection, and lung, kidney, spleen, cecal tonsils, bursa of Fabricius, trachea, pancreas, liver, heart, brain, duodenum, ileum, descending colon, and jejunum tissues were collected. The remaining chickens were observed for clinical signs of illness and lethality for 2 weeks. Virus titers were determined in eggs and expressed as log_10_ EID_50_/g of tissue. Data are expressed as *M* ± *SD*. Dashed black lines indicate the lower limit of detection. Differences were analyzed with a paired Student’s *t*-test and were considered statistically significant at ^∗^*p* < 0.05 compared to control.

To determine the pathogenicity of the viruses in chickens, we intranasally inoculated 6-week-old SPF white leghorn chickens with 10^5^ EID_50_ of either H5N6 virus (i.e., SW8 or ZH283). All inoculated chickens exhibited clinical signs of illness, including severe depression, cloudy eye, and intermittent head-shaking, and died within 5 DPI, with a mean death time (MDT) of 3.3 to 4.0 d (**Figure 1A**). SW8 and ZH283 replicated systemically in chickens and at 3 DPI was detectable in all tested organs, including the respiratory tract (i.e., lung and trachea), kidney, lymphoid tissues (i.e., spleen, cecal tonsils, and bursa of Fabricius), pancreas, liver, brain, intestinal tract (i.e., duodenum, ileum, descending colon, and jejunum), and heart. SW8 and ZH283 replicated efficiently in the lower respiratory tract; high viral titers were detected in the lung, with mean titers of 6.33 log_10_EID_50_ and 8.58 log_10_EID_50_, respectively (**Figure 1B**). The two novel viruses also replicated in the brain, spleen, and bursa of Fabricius, with mean titers of 4.83–7.17 log_10_EID_50_, 5.83–7.33 log_10_EID_50_, and 6.08–7.58 log_10_EID_50_, respectively (**Figure 1B**). Overall, the two novel H5N6 influenza viruses of wild bird origin showed high pathogenicity in chickens and could replicate systematically in them.

### Transmissibility of A(H5N6) Influenza Viruses of Wild Bird Origin in Chickens

**FIGURE 2:**
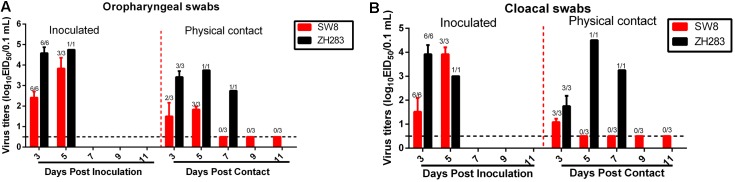
Direct contact transmissibility of H5N6 influenza viruses of wild bird origin among chickens. Viral titers of ZH283 and SW8 in oropharyngeal swabs **(A)** and cloacal swabs **(B)** in H5N6 influenza virus-inoculated and physical contact chickens. Three chickens were inoculated intranasally with 10^5^ EID_50_ of SW8 or ZH283, whereas three naïve chickens were placed in the cage of H5N6-infected chickens at 24 h post-infection to initiate contact. Oropharyngeal and cloacal swabs were collected from infected and naïve contact chickens at indicated time points; virus titers were titrated and are expressed as log_10_EID_50_/0.1 mL. Data are expressed as *M* ± *SD*. The proportion of chicken swabs presenting infectious virus from all detected swabs at indicated time points appears in the figure above each group. Dashed black lines indicate the lower limit of detection.

To evaluate the horizontal intraspecies transmissibility of the two novel H5N6 viruses, three SPF chickens were intranasally inoculated with 0.2 mL PBS and introduced into the same cage as a naïve contact group, which were then housed with chickens inoculated with SW8 or ZH283. Shedding of SW8 could be detected from both oropharyngeal and cloacal swabs within 3 DPI, with viral titers in the ranges of 2.42–3.83 log_10_EID_50_ in oropharyngeal swab samples and of 1.52–3.79 log_10_EID_50_ in cloacal swab samples. ZH283 could also be detected from oropharyngeal and cloacal swabs within 5 DPI, with viral titers in the range of 4.58–4.75 log_10_EID_50_ in oropharyngeal swabs and of 3.50–3.90 log_10_EID_50_ in cloacal swabs (**Figures 2A,B**). Naïve contact chickens co-housed with chickens inoculated with SW8 did not die during the observation time, but all contact group chickens seroconverted and exhibited high titers (9.33 ± 0.58 log2), as shown in **Table 2**. Viral shedding was observed in both oropharyngeal and cloacal swabs, and viral titers of 1.50–1.83 log_10_EID_50_ within 5 DPI were detected in oropharyngeal swabs (**Figure 2A**); however, viral titers of the cloacal swabs could be detected (1.08 log_10_EID_50_) at 3 DPI (**Figure 2B**). Naïve contact chickens co-housed with chickens inoculated with ZH283 exhibited 100% lethality and mortality, with a MDT of 5.0 days (**Table 2**), and exhibited clinical signs of illness, including coughing, cloudy eye, and dyspnea. All surviving chickens in the naïve contact group co-housed with ZH283-infected chickens shed virus from the oropharynx and cloaca within 7 DPI, with mean viral titers of 2.75–3.75 log_10_EID_50_ in oropharyngeal swabs and of 1.75–4.50 log_10_EID_50_ in cloacal swabs (**Figures 2A,B**). In short, results demonstrate that the two novel H5N6 influenza viruses replicated efficiently in chickens and exhibited efficient transmission via direct contact in the chicken model.

**Table 2:** Illness, mortality and HI titers of SPF chickens response to H5N6 influenza virus infection^a^.

Strains	Titer (log_10_EID_50_)	Group	Illness^b^	Mortality (%)	HI titer^c^ (log2, mean ± SD)	MDT
A/oriental magpie-robin/Guangdong/ SW8/2014 (H5N6)	7.88	Inoculated	3/3	3/3 (100)	–^d^	4.0
		Contact^e^	0/3	0/3	3/3 (9.33 ± 0.58)	
A/Pallas’s sandgrouse/ Guangdong/ZH283/2015 (H5N6)	8.50	Inoculated	3/3	3/3 (100)	–	3.3
		Contact	3/3	3/3 (100)	–	5.0
Controls (no virus exposure)			0/3	0/3	0/3	


*^a^Unless indicated otherwise, data represent the number of affected animals/animals in the group. Six-week-old chickens were inoculated by the intranasal route with 10^*5*^ EID_*50*_ of each virus in a 0.2 mL volume; HI, hemagglutination inhibition; MDT, mean death time.*

*^b^Severe depression, coughing, cloudy eye, dyspnea and intermittent head-shaking.*

*^c^HI titer was assayed in serum samples taken at 14 days post-inoculation. Data show the ratio of antibody-positive chickens to the number of virus-inoculated chickens.*

*^d^All the chickens died at the end of the observation.*

*^e^Three additional naïve contact chickens were placed with inoculated chickens as a contact group 24 h after inoculation.*

### Expression of TLRs and S1PR1 in the Target Tissues of H5N6-Infected Chickens

**FIGURE 3:**
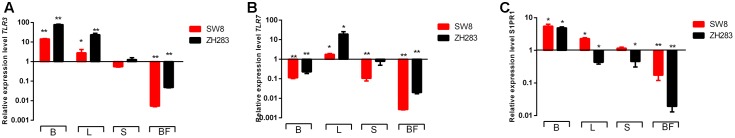
Toll-like receptors (TLRs) and sphingosine-1-phosphate-1 receptor (S1RP1) expression profiles in the target tissues of chickens infected with H5N6. At 3 days post-infection, the target tissues (i.e., brain, lung, spleen, and bursa of Fabricius) of H5N6-infected chickens were harvested for TLR and S1RP1 mRNA level detection via qRT-PCR method. **(A)** *TLR3*, **(B)** *TLR7*, **(C)** *S1PR1*. Data are expressed as *M* ± *SD*. Differences were analyzed with a paired Student’s *t*-test and were considered statistically significant at ^∗^*p* < 0.05, ^∗∗^*p* < 0.01 compared to control. B, Brain; L, Lung; S, Spleen; BF, Bursa of Fabricius.

Toll-like receptors are PRRs with a unique and essential physiological function in host immune systems activated by pathogen-associated molecular patterns (Medzhitov, 2001). Expression profiles of two PRRs—*TLR3* and *TLR7*—were examined in the target tissues of H5N6-infected chickens. As shown in **Figure 3A**, in contrast to mock-infected chickens, their expression level of *TLR3* in the brain and lung was significantly elevated when induced by both viruses, with a fold increase of 2.80–78.56 in the brain or lung. In the spleen, the expression level of *TLR3* was downregulated in response to SW8 infection, yet upregulated following infection with ZH283. In the bursa of Fabricius, the expression level of *TLR3* was markedly downregulated when induced by both viruses. The expression level of *TLR7* was upregulated in the lung when induced by SW8 or ZH283, by 1.79- and 19.41-fold, respectively. However, the expression level of *TLR7* in the brain, spleen, and bursa of Fabricius showed different expression patterns when induced by the viruses; *TLR7* expression level was downregulated when induced by both viruses compared to the control, with a fold change of 0.003–0.78 in all tested tissues except lung tissue. In particular, *TLR7* expression remained low and was no longer visible in the bursa of Fabricius when triggered by both viruses. Notably, the expression levels of *TLR3* and *TLR7* in target tissues induced by ZH283 were generally greater than those induced by SW8 (**Figures 3A,B**).

As an indispensable regulator of inflammation activation, S1PR1 plays a crucial role in immune cell trafficking and immune response (Rivera et al., 2008). When induced by SW8, S1PR1 expression was upregulated in the brain, lung, and spleen—by 5.51-, 2.29-, and 1.16-fold, respectively—but not in the bursa of Fabricius (0.17-fold). However, the expression level S1PR1 showed different tendencies when infected by ZH283. Unlike the expression level of TLR3 and TLR7, S1PR1 expression in the tested tissues after infection with ZH283 was lower than that in response to infection with SW8 (**Figure 3C**). Our data indicate that the engagement of PRRs and S1PR1 by the H5N6 influenza virus occurs in a tissue-dependent manner.

### Expression of Proinflammatory Cytokines and Chemokines in the Target Tissues of H5N6-Infected Chickens

**FIGURE 4:**
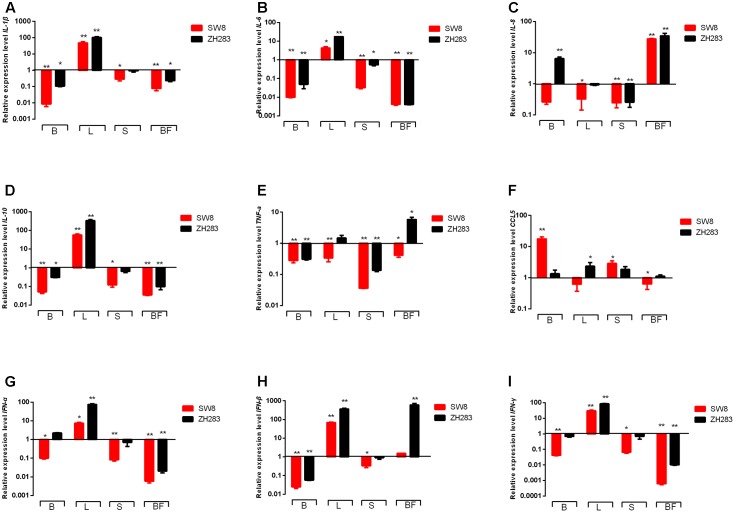
Proinflammatory cytokines and chemokines expression profiles in the target tissues of chickens when infected by H5N6. At 3 days post-infection, the target tissues (i.e., brain, lung, spleen, and bursa of Fabricius) of H5N6-infected chickens were harvested for proinflammatory cytokines and chemokines mRNA level detection via quantitative real-time polymerase chain reaction. **(A)** *IL-1*β, **(B)** *IL-6*, **(C)** *IL-8*, **(D)** *IL-10*, **(E)** *TNF-*α, **(F)** *CCL5*, **(G)** *IFN-*α, **(H)** *IFN-*β, **(I)** *IFN-*γ. Data are expressed as *M* ± *SD*. Differences were analyzed with a paired Student’s *t*-test and considered statistically significant at ^∗^*p* < 0.05, ^∗∗^*p* < 0.01 compared to the control. B, Brain; L, Lung; S, Spleen; BF, Bursa of Fabricius.

The engagement of TLRs by influenza virus in specific target tissues initiated animal immunity via the production of proinflammatory cytokines and chemokines, including *IL-1*β, *IL-6*, *IL-8*, *IL-10*, *TNF-*α, and *CCL5*. As shown in **Figures 4A,B,D**, the expression level of *IL-1*β, *IL-6*, and *IL-10* were remarkably unregulated in the lungs of tested chickens when infected by SW8 and ZH283 compared to those of mock-infected chickens. On the contrary, in the brain, spleen, and bursa of Fabricius, the expression levels of *IL-1*β, *IL-6*, and *IL-10* were downregulated when induced by both viruses. However, the expression levels of *IL-8*, *TNF-*α, and *CCL5* in the tested tissues of infected chickens showed a different expression patterns. As illustrated in **Figure 4F**, ZH283 induced an upregulated expression level of *CCL5* in all tested tissues, whereas SW8 induced an upregulated expression level of *CCL5* in the brain and spleen, but a downregulated one in the lung and bursa of Fabricius. Notably, ZH283-induced expression levels of *IL-1*β, *IL-8*, *TNF-*α, *IL-6*, and *IL-10* were greater than those induced by SW8 in all tested tissues of chickens (**Figures 4A–E**).

The activation of TLRs also mediated the activation of IFN regulatory factor 3/7, primarily by recruiting MyD88 or TNF receptor-associated factor 6, which ultimately activated I and II IFNs (i.e., *IFN-*α, *IFN-*β, and *IFN-*γ). In the lungs of tested chickens, both ZH283 and SW8 induced significantly upregulated expression levels of *IFN-*α, *IFN-*β, and *IFN-*γ by 7.55- and 75.97-fold, 68.23- and 362.80-fold, 30.11- and 85.31-fold, respectively (*p* < 0.05) compared to uninoculated chickens (**Figures 4G–I**). In contrast to the lung, the brain, spleen, and bursa of Fabricius showed different expression patterns in the levels of *IFN-*α, *IFN-*β, and *IFN-*γ in response to ZH283 and SW8 infection. However, ZH283 induced the expression levels of *IFN-*α, *IFN-*β, and *IFN-*γ to a greater extent than SW8 in the tested tissues of infected chickens.

In sum, our data indicate that the mRNA expression profiles of proinflammatory cytokines and chemokines showed different patterns in tested tissues likely associated with the pathogenic difference of both viruses in chickens.

### Expression of MHC Classes I and II Molecules in the Target Tissues of H5N6-Infected Chickens

**FIGURE 5:**
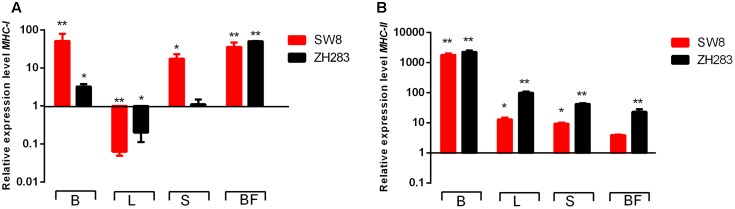
Major histocompatibility complex (MHC) classes I and II molecule expression profiles in the target tissues of chickens infected by H5N6. At 3 days post-infection, target tissues (i.e., brain, lung, spleen, and bursa of Fabricius) of H5N6-infected chickens were harvested for MHC classes I and II molecule mRNA level detection via quantitative real-time polymerase chain reaction. **(A)** *MHC-I*, **(B)** *MHC-II*. Data are expressed as *M* ± *SD*. Differences were analyzed with a paired Student’s *t*-test and considered statistically significant at ^∗^*p* < 0.05, ^∗∗^*p* < 0.01 compared to the control. B, Brain; L, Lung; S, Spleen; BF, Bursa of Fabricius.

To investigate whether MHC classes I and II molecules were involved in the host innate immune response to H5N6 influenza virus infection, we examined their expression levels in the lung, brain, spleen, and bursa of Fabricius in chickens at 3 DPI. As illustrated in **Figures 5A,B**, MHC classes I and II molecule expression levels were upregulated in the brain, spleen, and bursa of Fabricius when infected by both viruses. In the lung, in contrast to the mock-infected control, the expression level of the MHC class I molecule was remarkably downregulated (0.063- and 0.20-fold, respectively, *p* < 0.05); however, that of the MHC class II molecule was significantly upregulated when induced by SW8 and ZH283 (12.83- and 99.08-fold, respectively, *p* < 0.05). Those results demonstrated that MHC classes I and II molecules could play a significant role in the course of host innate immune response to H5N6 influenza virus infection in chickens.

## Discussion

The first case of human infection with H5N6 AIVs was reported in southwest China’s Sichuan Province in 2013 (Pan et al., 2016). Results of epidemiological surveillance show that the viruses have recently been isolated from humans (Shen et al., 2016), domestic poultry (Bi et al., 2015; Butler et al., 2016; Du et al., 2017; Li et al., 2017), pigs (Li et al., 2015), environmental samples (Yuan et al., 2016), cats (Yu et al., 2015), and wild birds (Bi et al., 2016b) and resulted in heavy losses in the poultry industry. However, the pathogenicity and transmissibility of H5N6 AIVs have remained unclear. In the current research, we systematically investigated the pathogenicity, transmissibility and the host immune-related gene in the target tissues of infected chickens when challenged by those of wild bird-origin H5N6 AIVs. Our findings provide insights into understanding the host innate immune response of chickens to infection with different pathogenicities of wild bird-origin H5N6 AIVs.

Importantly, we found that both H5N6 viruses isolated from wild birds were highly pathogenic and could efficiently be transmitted in chickens. Both viruses were shed from the oropharynx and cloaca in inoculated chickens and could be efficiently transmitted from infected chickens to naïve contact groups, the latter of which also shed viruses from both the cloaca and oropharynx throughout the experimental period. That the H5N6 HPAIVs isolated from wild birds could infect and be transmitted in chickens suggests that they may co-circulate in poultry and thus pose a great threat to the poultry industry.

Notably, chickens inoculated with SW8 showed high pathogenicity, whereas naïve contact chickens infected showed no deaths. By contrast, chickens inoculated with ZH283 showed high pathogenicity, with a mortality rate of 100% within 2–3 days and efficient horizontal transmission in chickens. The mechanisms of lethality and transmissibility might be associated with mutations at positions K52T, I155T, and A544V of the HA protein, at positions K207R and Y436H of the PB1 protein, and at position T515A of the PA protein (Hulse-Post et al., 2007; Li et al., 2014). However, with the exception of position I155T of the HA protein, no mutations were observed in ZH283, which suggests that differences in the pathogenicity and transmissibility of H5N6 influenza viruses in chickens correlate with the probability of their being at position I155T of the HA protein. In addition, the transmissibility of H5N6 AIV in different birds may also depend on the stability of viral particles and the difference of viral protein structure, relative humidity, and temperature (Webster et al., 1992; Lowen et al., 2007). However, our experiment posed several limitations, meaning that more viral strains isolated from different animals and species need to be tested in order to investigate the correlation between pathogenicity and host immunity. Further investigation is also clearly needed to elucidate the differences of pathogenicity, transmissibility, and host innate immune response to infection with H5N6 AIVs in chickens.

Remarkably, the expression levels of *TLR3* and *S1PR1* were upregulated in the brain following infection with SW8 and ZH283, yet showed different expression patterns in lymphoid tissues. Similarly, the production of *TLR7*, *IL-1*β, *IL-6*, *IL-10*, and *IFN-*γ were upregulated in the lung but downregulated in brain, spleen, and bursa of Fabricius in response to both viruses. Such results suggest that the engagement of the TLRs and cytokines are involved in a tissue-dependent manner. Previous studies have revealed tissue-specific immune responses following infection with H5N1 (Wei et al., 2013), H5N2 (Vanderven et al., 2012), and H7N1 (Cornelissen et al., 2012). The difference of cell types could be associated with immune responses and virus titers in the tissues tested for infection.

The robust production of proinflammatory cytokines and chemokines such as *IL-1*β, *IL-6*, *IL-8*, *IL-10*, *TNF-*α, *MCP-1*, *IFN-*α, *IFN-*β, and *IFN-*γ in mammals during influenza virus infection, referred to as *cytokine storms*, have been confirmed to contribute to the severity of pathological damage via immune-mediated mechanisms (Walsh et al., 2011; Teijaro et al., 2014). In our study, the expression levels of *IL-1*β, *IL-10*, and *IFN-*β in the lungs and *MHC-II* in the brain were upregulated to a remarkably high level after infection with ZH283 and SW8, although were greater for ZH283 than SW8. Moreover, the expression level of S1PR1 in tested tissues following infection with ZH283 was less than that following infection with SW8. Consistent with the results of other studies (Walsh et al., 2011; Teijaro et al., 2014), our results demonstrated that the activation of S1PR1 can suppress the induction of cytokines, chemokines, and PRRs, meaning reducing morbidity and mortality, in chickens infected with H5N6. However, the specific mechanism of action remains to be determined.

In sum, both H5N6 AIVs were highly pathogenic to chickens, caused multiple systemic infections in tissues, and were efficiently and rapidly transmitted in chickens. Those results indicate that H5N6 viruses could be transmitted to domestic poultry, which represents a serious threat to the poultry industry and both human and animal health. Furthermore, the expression profiles of PRRs, proinflammatory cytokines, chemokines, and MHC molecules in the tested tissues of H5N6-infected chickens were involved in a tissue-dependent manner. Lastly, our experiments demonstrated that ZH283 was associated with greater pathogenicity in chickens, for high virus titers appeared in tested tissues early in the infection process and were accompanied by the excessive expression of cytokines. Such data provide new insights into the relationship between the pathogenicity of H5N6 AIVs and host immune responses to them in chickens.

## Author Contributions

SG, YK, and TR designed the study. YK, SG, RY, HM, ZW, BX, XD, FW, JX, and ML contributed reagents/materials and performed the statistical analysis. YK analyzed the data. YK and SG wrote the manuscript.

## Conflict of Interest Statement

The authors declare that the research was conducted in the absence of any commercial or financial relationships that could be construed as a potential conflict of interest.

## Footnotes

1 http://www.who.int/en/

2 http://www.oie.int/fileadmin/Home/eng/Health_standards/tahm/2.03.04_AI.pdf

3 http://www.who.int/csr/don/4-january-2016-avian-influenza-china/en/
